# A reappraisal of successive negative contrast in two populations of domestic dogs

**DOI:** 10.1007/s10071-015-0947-0

**Published:** 2016-01-07

**Authors:** Stefanie Riemer, Sarah L. H. Ellis, Sian Ryan, Hannah Thompson, Oliver H. P. Burman

**Affiliations:** Animal Behaviour, Cognition and Welfare Research Group, School of Life Sciences, University of Lincoln, Joseph Banks Laboratories, Beevor Street, Lincoln, LN6 7DL UK

**Keywords:** Successive negative contrast (SNC), Dog *Canis familiaris*, Food reward, Reward sensitivity, Environment, Affective state

## Abstract

When an anticipated food reward is unexpectedly reduced in quality or quantity, many mammals show a successive negative contrast (SNC) effect, i.e. a reduction in instrumental or consummatory responses below the level shown by control animals that have only ever received the lower-value reward. SNC effects are believed to reflect an aversive emotional state, caused by the discrepancy between the expected and the actual reward. Furthermore, how animals respond to such discrepancy has been suggested to be a sign of animals’ background mood state. However, the occurrence and interpretation of SNC effects are not unequivocal, and there is a relative lack of studies conducted outside of laboratory conditions. Here, we tested two populations of domestic dogs (24 owned pet dogs and 21 dogs from rescue kennels) in a SNC paradigm following the methodology by Bentosela et al. (J Comp Psychol 123:125–130, 2009), using a design that allowed a within-, as well as a between-, subjects analysis. We found no evidence of a SNC effect in either population using a within- or between-subjects design. Indeed, the within-subjects analysis revealed a reverse SNC effect, with subjects in the shifted condition showing a significantly higher level of response, even after they received an unexpected reduction in reward quality. Using a within-, rather than a between-, subjects design may be beneficial in studies of SNC due to higher sensitivity and statistical power; however, order effects on subject performance need to be considered. These results suggest that this particular SNC paradigm may not be sufficiently robust to replicate easily in a range of environmental contexts and populations.

## Introduction

Many mammals will show a reduction in instrumental or consummatory responses when they experience an unexpected shift from a higher to a lower quality and/or quantity of reward, relative to a control group that is exposed only to the lower-level reward (Papini et al. 1988; Flaherty 1999; Mustaca et al. 2000; Bergvall et al. 2007; Catanese et al. 2011). If the responses of the ‘downshifted’ subjects fall below those of animals who have only ever received the less preferred reward (‘unshifted’ subjects), the phenomenon is known as a successive negative contrast (SNC) effect (see Flaherty 1999, for review). SNC effects have been found in some mammalian species, including in rats (*Rattus norvegicus*, e.g. Crespi 1942; Mellgren 1972; Pellegrini and Mustaca 2000), mice (*Mus musculus*, Mustaca et al. 2000), sheep (*Ovis aries*, Catanese et al. 2011; Greiveldinger et al. 2011), fallow deer (*Dama dama*, Bergvall et al. 2007), two marsupials (*Lutreolina crassicaudata* and *Didelphis albiventris*, Papini et al. 1988) and domestic dogs (*Canis familiaris*, Bentosela et al. 2009). With the exception of the starling (*Sturnus vulgaris*, Freidin et al. 2009), to date investigation of SNC in other vertebrate species has given negative results, for example, in pigeons (*Columba livia*, Papini 1997), toads (*Bufo arenarum*, Muzio et al. 1992; Papini et al. 1995), turtles (*Geoclemys reevesii*, Papini and Ishida 1994) and goldfish (*Carassius auratus*, Lowes and Bitterman 1967; Couvillon and Bitterman 1985). However, the effect has been observed in honey bees (Bitterman 1976; Wiegmann and Smith 2009) and bumble bees (Waldron et al. 2005).

The exaggerated change in behavioural response (e.g. reduced operant behaviour or reward consumption) as a result of an unexpected reduction in reward value implies that animals form reward expectations and compare the quantity or quality of the present reward with those received previously (Flaherty 1999), and it is suggested that they experience short-term aversive emotions (i.e. brief, transient and stimulus-dependent affective states, e.g. ‘disappointment’) if these expectations are not met (reviewed in Rosas et al. 2007; Justel et al. 2014; Papini 2014). How animals respond to such an unexpected reward reduction, in terms of either the strength or duration of their response, may also be dependent on their longer-term mood (i.e. enduring and stimulus-independent affective states, such as ‘depression’). Consequently, it has been suggested that SNC might be valuable as a way of informing us about the background affective state of subjects as well as directly inducing affect (Burman et al. 2008; Mitchell et al. 2012). Accordingly, rats of strains selected for high emotional reactivity show enhanced SNC effects (e.g. Cuenya et al. 2012; Freet et al. 2006; Gómez et al. 2009; Ortega et al. 2014; Rosas et al. 2007).

Burman et al. (2008), Mitchell et al. (2012) and Chaby et al. (2013) tested the effect of environmental manipulations on SNC in laboratory rats. In Burman et al. (2008), rats from unenriched housing, assumed to be experiencing a more negative affective state, displayed a prolonged response, expressed as slower running speeds in a runway, to an unexpected decrease in reward quantity compared to enriched rats, indicating enhanced sensitivity to reward loss (Burman et al. 2008). Along similar lines, rats that had experienced unpredictable and stressful environments during adolescence responded more strongly to a reward downshift than a control group in Chaby et al. (2013). However, Mitchell et al. (2012) found that rats kept in barren environments showed an attenuated SNC effect compared to individuals from enriched housing (Mitchell et al. 2012). The authors suggested that the apparent contradiction in results compared to the study by Burman et al. (2008) and Chaby et al. (2013) could be explained by the possibility that access to the test chamber itself (and the contrast to their unenriched home environment) induced a positive affective state in the unenriched subjects, since they had experienced daily reward-based training in this location. This reflects findings in other studies using cognitive approaches to assess affective state in animals, in which the affective state at the moment of testing may differ from that predicted a priori (e.g. Doyle et al. 2010; Burman et al. 2011). Thus, the occurrence and interpretation of SNC effects are not unequivocal, and more work is needed to validate its robustness as a measure of affective state in animals across different environments.

While SNC has been extensively investigated in the laboratory rat, less work has investigated responses to reward downshifts in other mammals (reviewed above). With few exceptions, the tested individuals came from highly standardised laboratory conditions, and it is thus questionable whether results can be generalised to more heterogeneous environments outside of the laboratory.

For a number of reasons, the domestic dog is a particularly valuable model species in which to advance the study of SNC as a measure of affective state. Firstly, dogs are a remarkably varied species residing in an extensive range of environments (Taylor and Mills 2007). For example, purpose-bred laboratory dogs represent a homogenous population residing in a homogeneous environment, whereas owned pet dogs represent a heterogeneous population residing in a heterogeneous environment. Secondly, numerous studies have demonstrated advanced cognitive abilities in the domestic dog (reviewed in Bensky et al. 2013), thereby making it a useful species to investigate SNC where experimental paradigms often involve elements of training. Finally, many dogs find themselves living temporarily or permanently in kennels, an environment which can result in poor welfare and negative affect (Hennessy et al. 1998; Coppola et al. 2006; Taylor and Mills 2007) due to a number of factors such as physical confinement (Wells 2004) and limited intra- and inter-specific contact, for example isolation from a former attachment figure (Tuber et al. 1999). Such an environment therefore provides an opportunity to extend previous findings of the influence of affective manipulations on SNC (e.g. Burman et al. 2008) to outside the laboratory, thereby further validating its reliability as a measure of affective state and, ultimately, animal welfare.

A pioneering study on SNC in domestic dogs, conducted by Bentosela et al. (2009), involved training pet (owned) dogs to receive a food reward in return for directing their gaze towards the experimenter’s eyes. One group of dogs were shifted from the high value food reward (dried liver) to the low value reward (dog food pellets), while the other group always received the low value food. Duration of eye gaze and proportion of food rejections in the two shift groups were compared. While the effect of shift condition on duration of eye gaze was not significant, Bentosela et al.’s (2009) results did suggest the occurrence of a SNC effect as manifested in the observed rate of food rejection. Conversely, Pongrácz et al. (2013) found no evidence of an incentive contrast effect when pet dogs were switched from sausage as a reward to carrot in a pointing task.

Thus, previous findings regarding the occurrence of a SNC effect in domestic dogs are inconclusive. Dogs’ strong reliance on human actions (Topál et al. 1997; Udell and Wynne 2008) or their interpretation of the pointing gesture as a command (e.g. Prato-Previde et al. 2008, but see Scheider et al. 2013) may account for the lack of a SNC effect in the study by Pongrácz et al. (2013). Given the small sample size in Bentosela et al. (2009) (13 subjects, 7 in the shifted group and 6 in the unshifted group), it is possible that clearer effects would emerge with a larger sample size due to higher statistical power. It is also conceivable that random variation in their between-subjects design may have reduced the effect, given the large inter-individual variability in both duration of eye gaze and food rejection rates. This could be addressed by conducting the experiment using a within-subjects design so that each subject acts as its own control (c.f. Keren and Lewis 2014).

The objectives of this study were therefore to confirm the findings of Bentosela et al. (2009) by using their methodology to test a population of owned (pet) dogs, but utilising an experimental design that allowed both a within-, as well as a between-, subjects comparison. In addition, because rescue and owned dogs may not necessarily respond to human interaction in the same way (e.g. Udell and Wynne 2008), we also applied the approach designed by Bentosela et al. (2009) to a population of dogs housed in rescue kennels.

## Methods

We tested two populations of dogs, owned dogs and dogs in rescue shelters, in a SNC task following the methodology by Bentosela et al. (2009). Nearly all dogs were tested twice, receiving each treatment (shifted/unshifted) in a randomised order, so that data could be analysed both at the between-subjects and at the within-subjects level.

### Food rewards

While the methodology by Bentosela et al. (2009) was followed as closely as possible, a different high value incentive (sausage) was used in the current study, as pilot studies revealed that some dogs rejected the dried liver as used by Bentosela et al. (2009). As for Bentosela et al. (2009), the dried food usually eaten by the dogs was used as the low value incentive for the owned dogs. Since the kennelled dogs were fed a variety of dry food brands, a commercially available wholegrain mixer was used as the low value incentive. To ensure that the assumed higher value reward was indeed preferred by the dogs, a subset of dogs in our study were tested in a food preference test (Ellis et al. 2014). Due to a lack of availability, not all dogs could be exposed to the preference test.

### Food preference test

Eighteen dogs participating in the SNC experiment (nine owned pet dogs and nine rescue dogs from Mayflower Sanctuary, South Yorkshire, UK; see section ‘Subjects’ below for demographic details) were tested in the food preference test, according to the methodology outlined in Ellis et al. (2014). Following sampling of one piece of each food type, two bowls, one containing a piece of sausage and the other a piece of dry food, were placed under two separate wire covers, rendering them visible, but inaccessible, to the dogs. The dog was then released and the total amount of time spent investigating each inaccessible food bowl within a 1-min period was recorded.

### Subjects

Twenty-four owned pet dogs (*C. familiaris,* 13 females and 11 males ranging in age from 1 to 11 years) and 21 rescue dogs (10 males and 11 females ranging in age from 10 months to 10 years, data on neuter status not collected) completed the study. Both populations included various breeds and breed crosses. Owned dogs were recruited from private homes located in the Lincolnshire, UK, area. Rescue dogs came from the South Lincolnshire Centre of Jerry Green Dog Rescue, UK (12 subjects) and the Mayflower Sanctuary, South Yorkshire, UK (9 subjects). Rescue dogs’ length of stay at the shelter ranged from 2 days to more than 2 years. Dogs were housed singly in kennels, but these were rotated occasionally so that access to outdoor runs was available at times. Additionally, most dogs were taken on walks by volunteer dog walkers daily or had the opportunity to run off-lead in a fenced enclosure. Dogs were excluded from the study if they met any of the following criteria: (1) present or past resource guarders (e.g. guarding of their food bowl); (2) history of serious human-directed aggression; (3) injury and/or illness; (4) in oestrous or lactating at the time of study: (5) appeared to be too anxious around the unfamiliar experimenter or in the test environment; and (6) failed to eat one of the reward types. Ten owned dogs and 14 rescue dogs were excluded on this basis; they are not included in the sample numbers given above.

### Procedure

Owned dogs were tested in a familiar room within their homes, while kennelled dogs were tested in a room at the rescue shelter to which they were all habituated prior to testing by giving them free off leash access to the room. Both groups of dogs had water available ad libitum and were fed twice daily (morning and evening) on non-testing days. Owners and shelter staff were asked not to feed the dogs on the morning of the testing day.

Dogs were required to direct their eye gaze to the experimenter’s eyes for one second at a time for a food reward following the methodology as described in Bentosela et al. (2009). Thirteen trials of 2 min each were conducted, with inter-trial intervals of 2 min. During breaks between trials, the experimenter left the test room so that the dog could not continue offering eye contact.

There was no pre-training, but to facilitate learning of the task during the first trial, the dog’s name was called once and any eye contact was rewarded immediately. In trials 2–13, dogs were rewarded only for an eye gaze of one second duration directed towards the experimenter. Pilot work revealed that the kennelled dogs struggled to understand what they were being reinforced for in the absence of a verbal marker. Therefore, in addition to the methodology by Bentosela et al. (2009), the experimenter verbally marked eye contact with the word ‘Good’ prior to giving the food to the dog. The use of the verbal marker allowed the dogs to learn the desired behaviour more quickly, preventing either the need for additional training or deviation from the number of trials previously used in Bentosela et al.’s (2009) study. To ensure consistency, we also used the verbal marker with the owned dogs. Furthermore, due to the nature of the testing rooms, it was not possible to store the food rewards in a container located on a tall table as described in Bentosela et al.’s (2009) study. Instead, food rewards were stored within separate containers within a single pouch worn around the experimenter’s waist.

The food reward given in each trial depended on the treatment to which the dog was randomly assigned, unshifted (12 owned dogs, 11 rescue dogs) and shifted (12 owned dogs, 10 rescue dogs). In the unshifted treatment, dogs received the low value reward throughout the entire study (13 trials). In the shifted treatment, dogs received the high value reward during the first eight trials (pre-shift trials) and were then given the low value food reward for trials 9–12 (post-shift trials), before receiving the high value food reward once more on the final trial (re-shift trial). This change back to high value food reward in trial 13 for the shifted treatment was to confirm that any reduction in eye gazes and associated food consumption seen in the post-shift trials was not due to satiation (see Bentosela et al. 2009).

For the owned dogs, three female experimenters shared the testing, and testing of kennelled dogs was performed by one of the two experimenters. For 16 of the 25 owned dogs and all of the kennelled dogs, an observer (female) was also present who recorded duration of eye gaze and number of food rejections. For the kennelled dogs, the observer stayed with the dogs during test breaks, during which the experimenters left the room. All trials were video-recorded, and for trials without an observer, duration of eye gaze and number of food rejections were later coded from the videos. Correspondence between video and live recordings was assessed on the basis of 15 randomly selected videos and was 100 % for both duration of eye gaze and the number of food rejections.

Approximately 1 week after the first test, subjects were re-tested (with the exception of one kennelled dog and one owned dog who were not available for re-testing) using an identical test protocol, with the treatments swapped. Thus, data could be analysed not only at the between-subjects level, as in Bentosela et al. (2009), but also as a within-subjects comparison, with each dog acting as its own control.

### Data analysis

Statistical analyses were conducted with IBM SPSS version 21.0 and SISA (http://www.quantitativeskills.com/sisa/statistics/fisher.htm), with the alpha value set at the 0.05 level. All analyses involved two-tailed tests and data satisfied the assumptions of parametric testing. For descriptive statistics, mean and standard error are indicated. The food preference test was analysed using a repeated measures general linear model (GLM), with time spent investigating the food as dependent variable and the predictors food type (within-subjects factor) and population (owned/rescue dogs, between-subjects factor).

The SNC experiment was similarly analysed with GLMs. We analysed two dependent measures per trial (as utilised in Bentosela et al. 2009): gaze duration (calculated from the number of one second eye gazes) and proportion of food rejection. For the between-subjects design, repeated measures GLMs were calculated for pre-shift trials (trials 1–8) and post-shift trials (trials 9–12), with trial as a within-subjects factor and treatment (shifted/unshifted) and population (owned/rescue dogs) as between-subjects factors. Trial 13 (re-shift) was analysed using a GLM with treatment and population as between-subjects factors. A Fisher’s exact test [two-sided, mid-*p* (see Lydersen et al. 2009)] was calculated to compare the number of individuals of both treatment groups showing food rejection in at least one of the trials of the post-shift phase. Due to the rarity of occurrence, data on food rejection rates were not amenable to modelling, and data for owned and rescue dogs were combined.

For the within-subjects analysis of the experimental design (available for 23 owned pet dogs and 20 rescue dogs), univariate mixed effect GLMs were fitted with treatment (shifted/unshifted), trial, population (owned/rescue) and test order (i.e. which treatment dogs received first) as fixed factors, and dogID as a random factor. For trial 13, a GLM was fitted with treatment, population and test order as fixed factors, and dogID as a random factor. Selection of terms in the model specification was determined by a priori predictions. The subsequent model was simplified with non-significant terms removed using a stepwise backward simplification (Calcagno et al. 2010). Interactions were investigated post hoc using either independent or paired *t* tests as appropriate.

## Results

### Preference test

Of 18 tested dogs, 15 spent a greater amount of time investigating the inaccessible sausage compared to the inaccessible dry food. This was also reflected in investigation times: dogs spent significantly more time (mean 22.24 ± 3.47 s) investigating the inaccessible sausage compared to the inaccessible dry food (mean 8.08 ± 1.24 s; *F*_1,16_ = 14.603, *P* = 0.002). There was a significant effect of population (*F*_1,16_ = 5.052, *P* = 0.039), with rescue dogs investigating more, regardless of food type, compared to owned dogs. There was no significant interaction between population and food type (*P* > 0.05).

### Between-subjects analysis: eye gaze

**Fig. 1:**
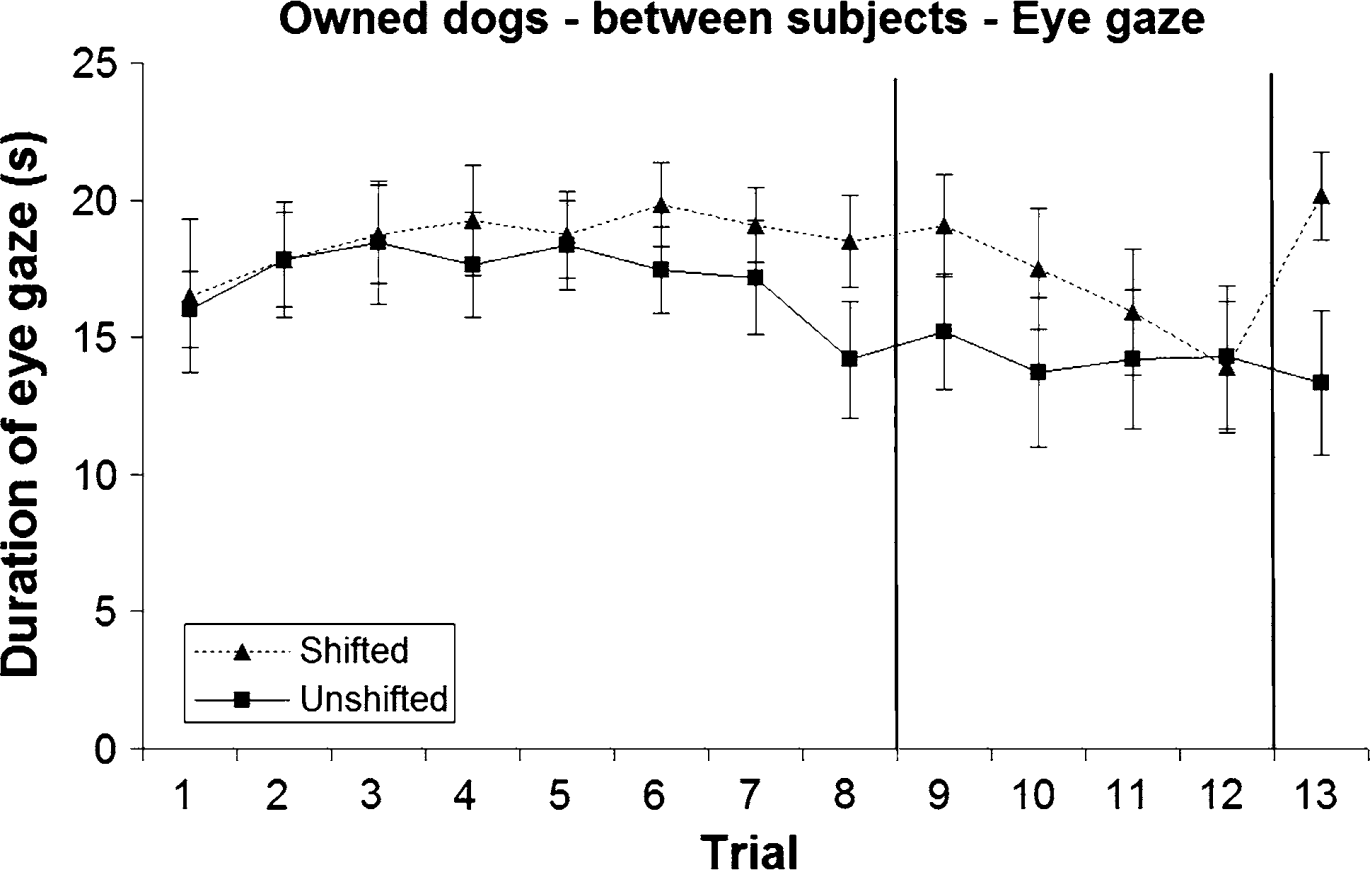
Mean and standard error of duration of eye gaze during the two treatments in the owned dogs (between-subjects analysis)

**Fig. 2:**
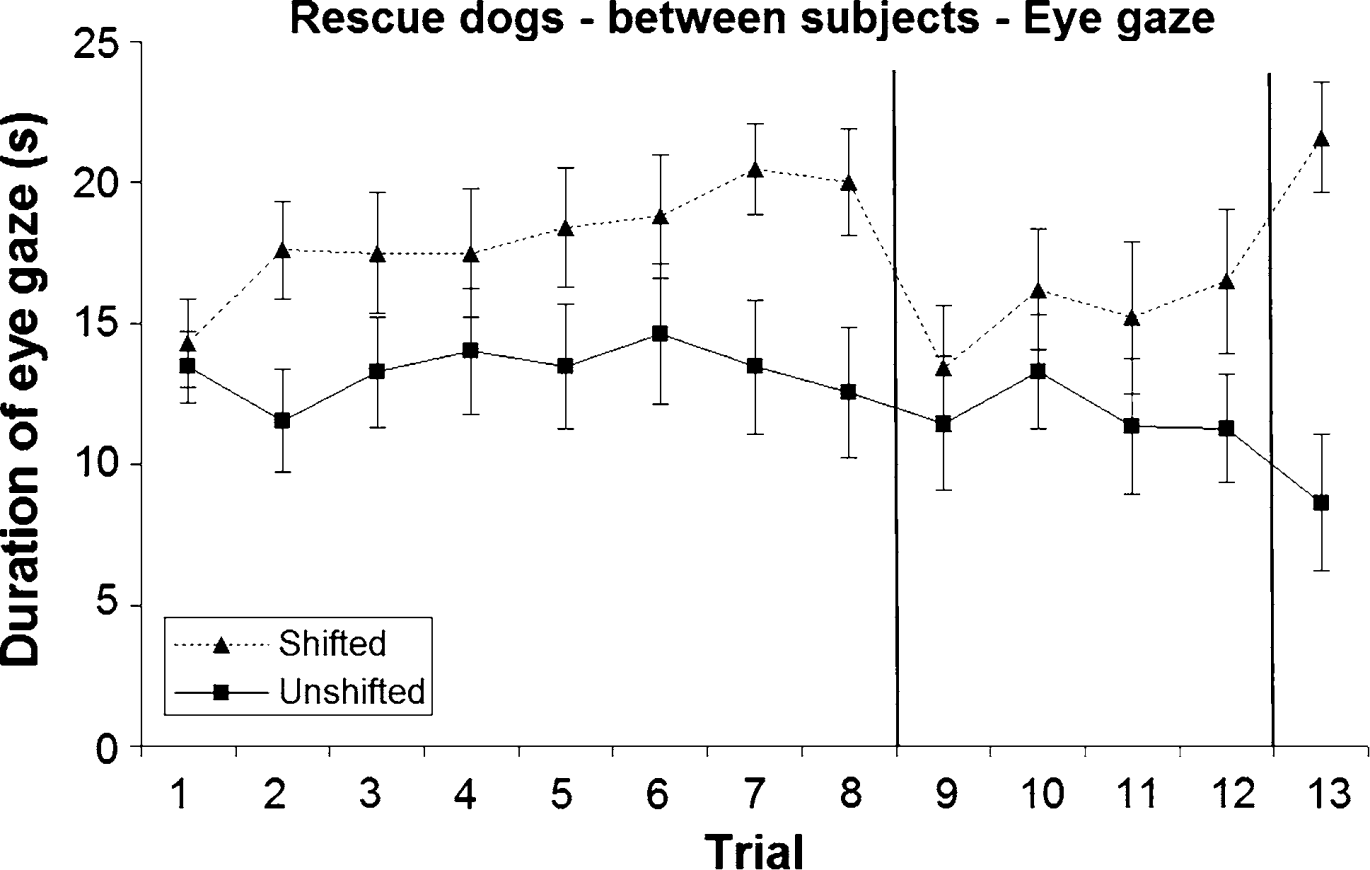
Mean and standard error of duration of eye gaze during the two treatments in the rescue dogs (between-subjects analysis)

Figures 1 and 2, respectively, show the duration of eye gaze by owned and rescue dogs.

#### Pre-shift trials

All dogs appeared to be more strongly incentivised by the sausage compared to the dry food in the initial eight trials, as indicated by a higher duration of eye gaze during the shifted treatment (mean 18.34 ± 0.47 s) compared to the unshifted treatment (mean 15.22 ± 0.51 s; Figs. 1, 2). Nonetheless, the effect of treatment and all other main effects and interactions were not statistically significant (*P* > 0.05).

#### Post-shift trials

Dogs showed no successive negative contrast effect, as there was no significant difference (*P* > 0.05) in mean gaze duration between treatments (shifted 13.09 ± 0.80 s; unshifted 12.52 ± 0.86 s) during post-shift trials 9–12, when all dogs received dry food as a reward (Figs. 1, 2). There was a significant trial × population interaction (*F*_3,123_ = 31.619, *P* = 0.020), reflecting overall higher responses by the owned dogs compared to rescue dogs, particularly during trial 9 (*t*_43_ = 1.937, *P* = 0.059). There were no other significant effects (*P* > 0.05).

#### Re-shift trial

The higher incentive value of the sausage compared to the dry food was demonstrated very clearly in the last trial, when dogs in the shifted group were re-shifted to sausage again, and showed a significantly greater duration of eye gaze (mean 20.81 ± 1.23 s) compared to dogs in the unshifted group (mean 11.09 ± 1.82 s; *F*_1,41_ = 20.140, *P* < 0.001; Figs. 1, 2). There were no other significant differences (*P* > 0.05).

### Between-subjects analysis: food rejection

**Fig. 3:**
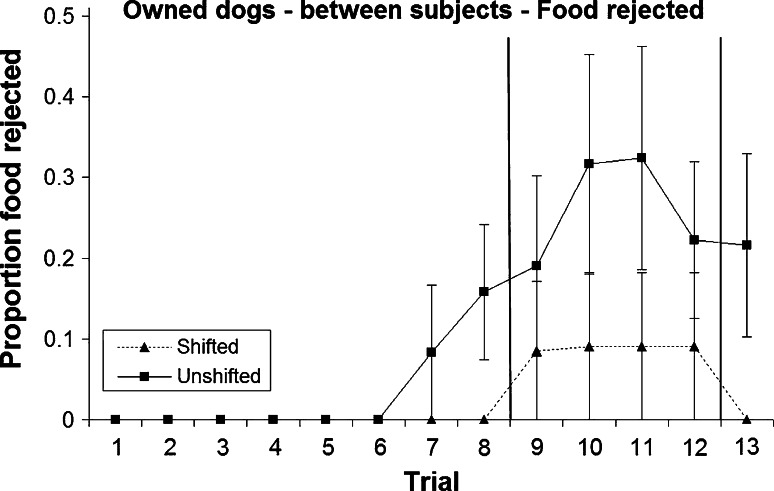
Mean and standard error of proportion of food rejected during the two treatments in the owned dogs (between-subjects analysis)

**Fig. 4:**
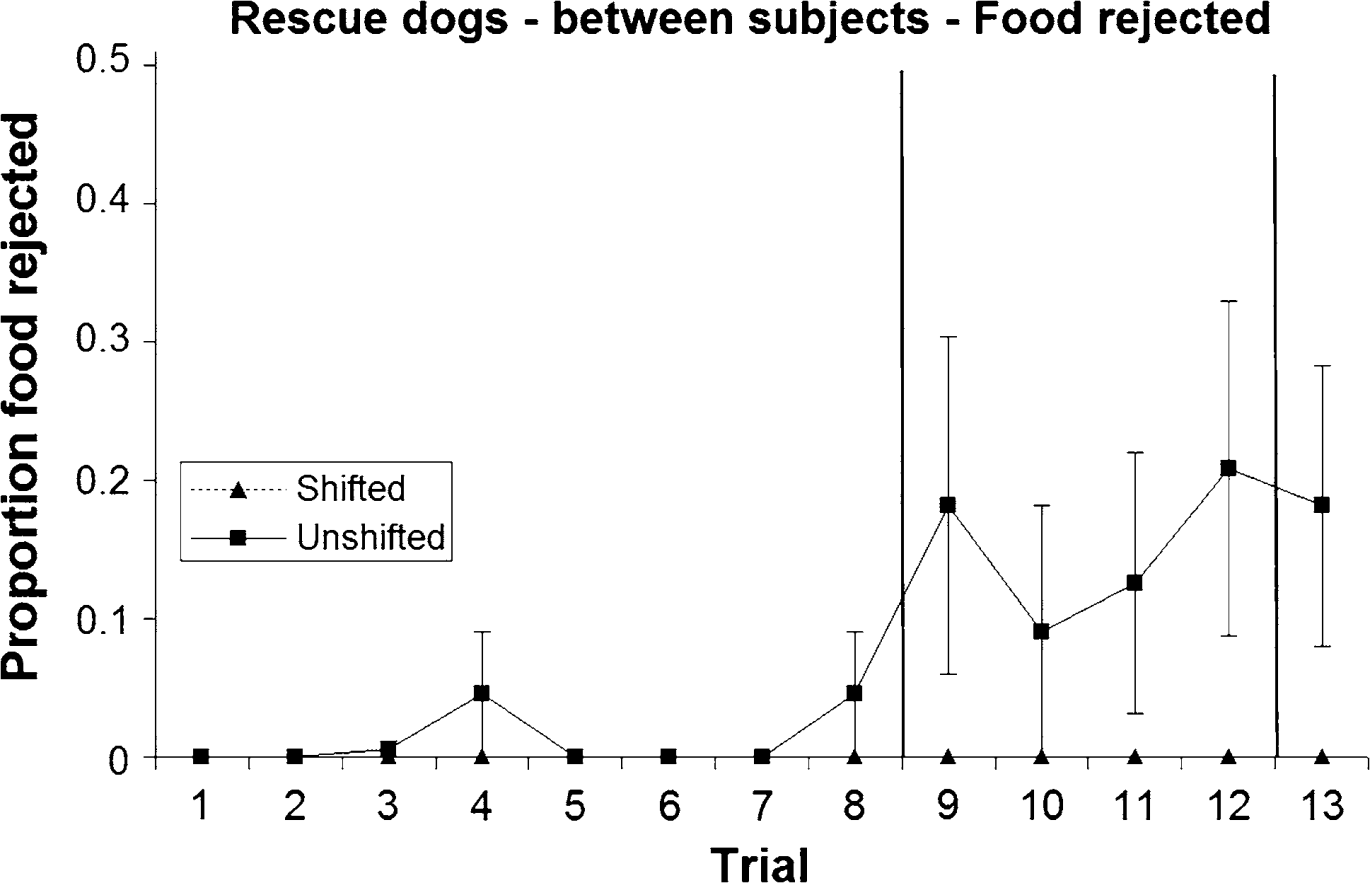
Mean and standard error of proportion of food rejected during the two treatments in the rescue dogs (between-subjects analysis)

Rejection of food was rare (overall, only 40 of 585 trials, 6.9 %), and during any one phase (pre-, post- or re-shift), no more than six dogs showed any food rejection at all (Figs. 3, 4; Table 1). Unlike unshifted dogs, dogs in the shifted treatment did not reject any food during pre- and re-shift trials; thus all rejections were of dry food, further confirming dogs’ preference for the sausage over the kibble. This difference tended towards significance during the pre-shift trials and was significant for the re-shift trial (Table 1). Post-shift rejection—although more common in the unshifted treatment—did not differ significantly between shift conditions. With the exception of one dog, all food rejections occurred from trial 7 onwards.

**Table 1:** Number of dogs rejecting at least one piece of food during pre-shift, post-shift and re-shift trials, respectively, and results of a Fisher’s exact test (mid-*p*) testing for differences between treatments (between-subjects analysis)

	Pre-shift	Post-shift	Re-shift
Number of shifted dogs showing food rejection	0	3	0
Number of unshifted dogs showing food rejection	4	6	6
Fisher’s exact test, mid-*p*	0.07	0.37	0.02

### Within-subjects analysis: eye gaze

#### Pre-shift

**Fig. 5:**
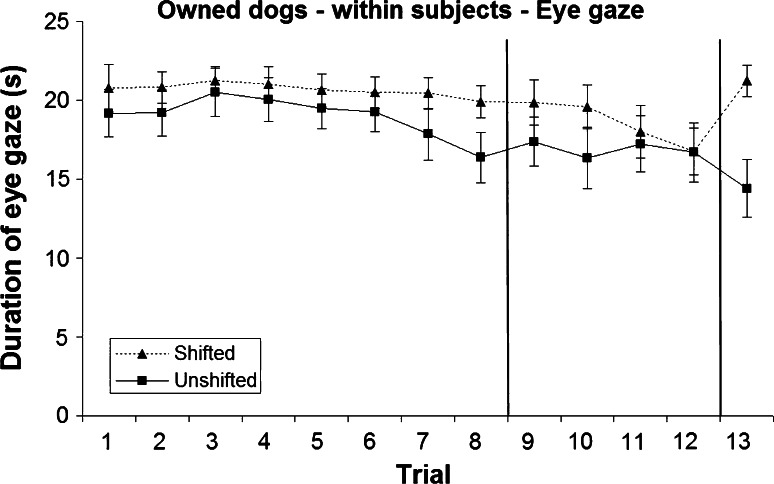
Mean and standard error of duration of eye gaze during the two treatments in the owned dogs (within-subjects analysis)

**Fig. 6:**
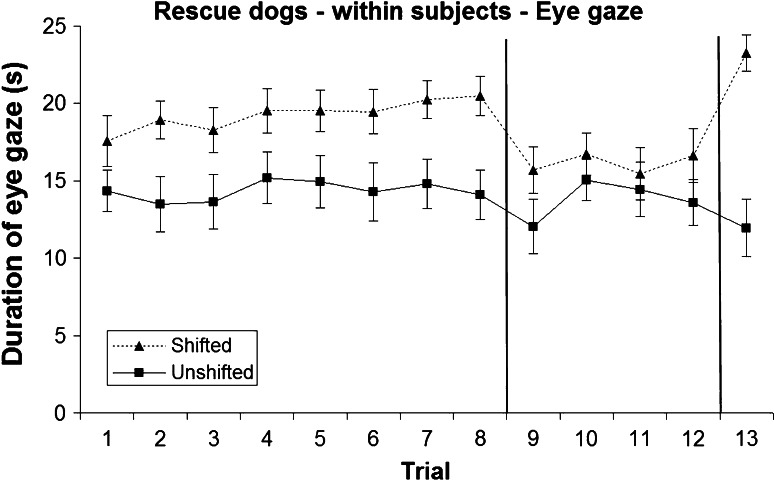
Mean and standard error of duration of eye gaze during the two treatments in the rescue dogs (within-subjects analysis)

Dogs showed a higher duration of eye gaze in the shifted treatment when they received sausage as a reward (mean 19.93 ± 0.30 s) than in the unshifted treatment (dry food, mean 16.74 ± 0.40 s; Figs. 5, 6), and there was a significant treatment × test order interaction (*F*_1,635_ = 70.273, *P* < 0.001). This indicated that for both test orders (unshifted first *t*_22_ = −5.79, *P* < 0.001; shifted first *t*_19_ = −2.822, *P* = 0.011), dogs gazed more when receiving the shifted (sausage) treatment than when receiving the unshifted (dry food) treatment, but that this effect differed in magnitude, with a greater difference in dogs that received the unshifted treatment first.

A significant treatment × population interaction (*F*_1,635_ = 20.384, *P* < 0.001) revealed that both owned and rescue dogs showed a longer duration of eye gaze during the shifted treatment (sausage) than during the unshifted treatment (dry food) across all pre-shift trials (owned *t*_22_ = −3.03, *P* = 0.006; rescue *t*_19_ = −6.582, *P* < 0.001; Figs. 5, 6), but that this was more pronounced in the rescue dogs. All other effects were non-significant (*P* > 0.05).

#### Post-shift

Although dogs in both treatments received dry food in trials 9–12, dogs still exhibited a longer duration of eye gaze during the shifted treatment (mean 17.32 ± 0.54 s) than during the unshifted treatment (mean 15.38 ± 0.60 s; Figs. 5, 6) revealing an apparent ‘reverse SNC effect’. A significant treatment × test order interaction emerged (*F*_1,296_ = 38.494, *P* < 0.001): dogs that received the unshifted treatment first gazed more when receiving the shifted treatment than when receiving the unshifted treatment (*t*_22_ = −4.422, *P* < 0.001), whereas those dogs that experienced the shifted treatment first gazed for similar durations regardless of treatment (*t*_19_ = 1.006, *P* = 0.327). No other effects were significant (*P* > 0.05).

#### Re-shift

When dogs were rewarded with sausage (shifted treatment), again they showed a much longer duration of eye gaze (mean 21.58 ± 1.24 s) compared to dogs receiving dry food (unshifted treatment, mean 13.04 ± 1.83 s; Figs. 5, 6), and there was a significant treatment × test order interaction (*F*_1,41_ = 4.104, *P* = 0.049). As for the pre-shift phase, this indicated that for both test orders (unshifted first *t*_22_ = −5.577, *P* < 0.001; shifted first *t*_19_ = −3.481, *P* = 0.003), dogs gazed more when receiving the shifted (sausage) treatment than when receiving the unshifted (dry food) treatment, with a greater magnitude of difference in dogs that received the unshifted treatment first. There were no other significant differences (*P* > 0.05).

### Within-subjects analysis: food rejection

**Fig. 7:**
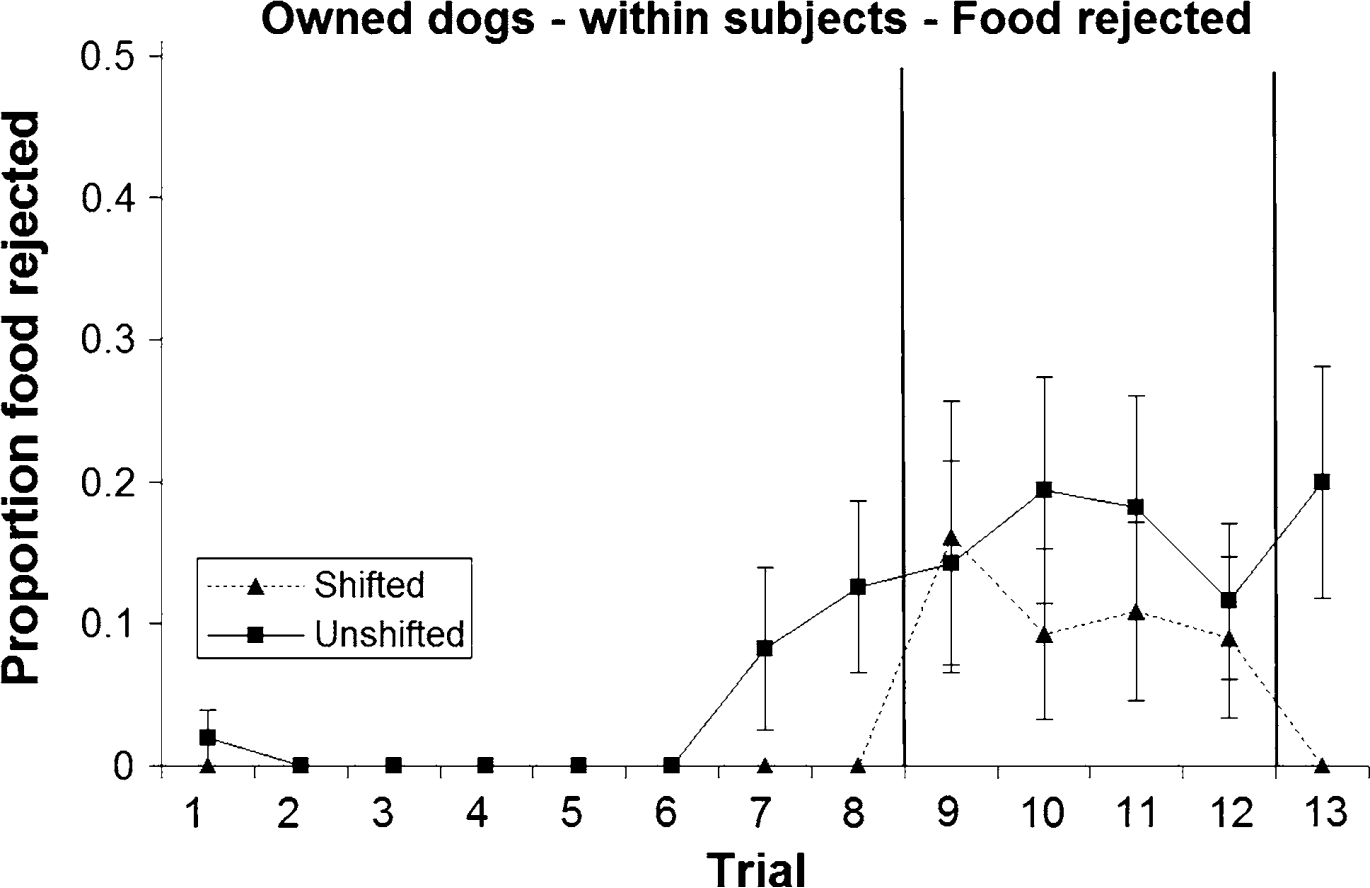
Mean and standard error of proportion of food rejected during the two treatments in the owned dogs (within-subjects analysis)

**Fig. 8:**
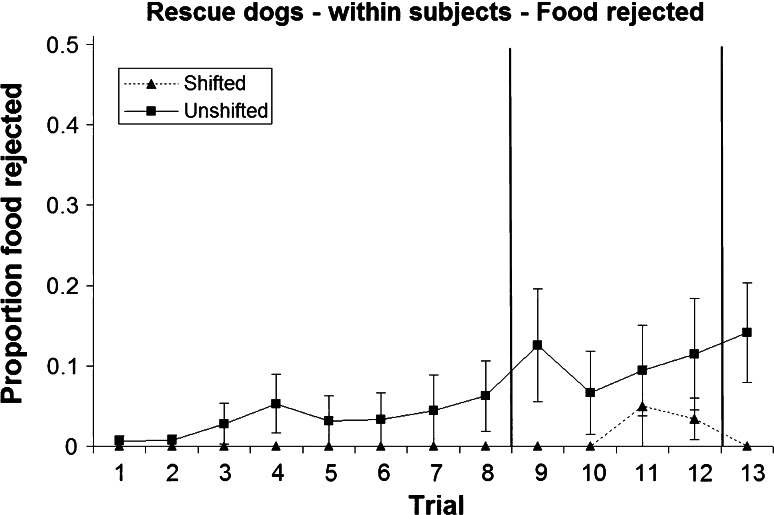
Mean and standard error of proportion of food rejected during the two treatments in the rescue dogs (within-subjects analysis)

Food rejection was rare, with only six of the 43 subjects rejecting any food at all during the shifted treatment (16.3 %) and 11 dogs during the unshifted treatment (25.6 %). During pre-shift and re-shift trials, none of the shifted dogs showed any food rejection, and a significantly higher number of unshifted dogs rejected at least one piece of food in comparison (Table 2; Figs. 7, 8). Following the downshift, six shifted dogs and ten unshifted dogs rejected food, which does not constitute a statistically significant difference (Table 2). Overall, the proportion of food rejected was minimal (57 of 1118 trials = 5.1 %; Figs. 7, 8).

**Table 2:** Number of dogs rejecting at least one piece of food during pre-shift, post-shift and re-shift trials, respectively, and results of a Fisher’s exact test (mid-*p*) testing for differences between treatments (within-subjects analysis)

	Pre-shift	Post-shift	Re-shift
Number of shifted dogs showing food rejection	0	6	0
Number of unshifted dogs showing food rejection	6	10	11
Fisher’s exact test, mid-*p*	0.02	0.35	<0.001

## Discussion

Dogs from both the owned and the rescue populations showed a preference for the sausage (high value reward) over the dry food (low value reward) in the food preference test, confirming the ascribed contingency values of the two food rewards (Ellis et al. 2014). This initial preference was reflected in the pre-shift (within-subjects analysis only) and re-shift trials (both between- and within-subjects analysis) during subsequent testing: dogs showed higher durations of eye gazing when rewarded with sausage compared to dry food, and rejections occurred exclusively with dry food.

Nonetheless, we could not replicate the finding by Bentosela et al. (2009) that dogs showed an SNC effect expressed in the proportion of food rejection. Notably, food rejection rates were far lower in our study (max. 30 % in a single trial) compared to Bentosela et al. (2009; up to 80 % for individual trials). It is possible that the difference in high value reward contributed to this difference in rejection rates between our study (sausage) and Bentosela et al. (2009) (dried liver), as findings in rats indicate that strength of SNC effect is related to the difference in hedonic value of the high- and the low-quality reward (Di Lollo and Beez 1966; Flaherty 1982, 1999; Catanese et al. 2011). Our observed lack of SNC effect could therefore indicate that the disparity in reward quality (high vs. low) was not great enough. However, this is unlikely given the preference demonstrated in both the initial preference test and in pre-shift and re-shift trials (notably, Bentosela et al. 2009 did not find a significant pre-shift difference).

As previous experience with the low value reward has been found to eliminate the SNC effect (Flaherty 1999), one could speculate that using the owned dogs’ own dry food as the low value reward contributed to the lack of SNC effect observed in our study. However, Bentosela et al. (2009) report a SNC effect using the same methodology. Moreover, for the kennelled dogs in our study, the dry food used was not their normal food, ruling this out further. It is also possible that populations in the two countries (Argentina and UK) differed with regard to training level and social experience. Previous studies have demonstrated behavioural differences in dogs from different countries on measures such as trainability, aggressiveness and reactivity (Bradshaw and Goodwin 1999; Takeuchi and Mori 2006; Notari and Goodwin 2007), highlighting the possibility that dogs residing in Argentina (Bentosela et al. 2009) and the UK (current study) may not behave in the same way in certain situations.

We also did not find an SNC effect in the operant response, as eye gaze duration in downshifted dogs never dropped below that shown in the unshifted treatment. Instead, we observed a reverse SNC effect (e.g. Papini 2014) in the within-subjects analysis, i.e. dogs that had received the high value reward during pre-shift trials maintained a significantly higher level of responses even after the reward downshift compared to dogs that received the same low value reward during all trials. This was unexpected, given that reverse SNC effects are suggested to be typical of non-mammalian vertebrates (Papini 2014). Thus, it has been theorised that mammals and non-mammalian vertebrates respond in fundamentally different ways to reward downshifts, with only mammals reacting ‘emotionally’ when anticipated rewards are not in line with their expectations (Muzio et al. 2011; but see Freidin et al. 2009). Reptiles, amphibians and fish typically show a reverse SNC effect by gradually adjusting level of responses to the lower reward quality (Lowes and Bitterman 1967; Couvillon and Bitterman 1985; Muzio et al. 1992; Papini and Ishida 1994; Papini et al. 1995; Muzio et al. 2011; Papini 2014), suggesting habit learning rather than incentive learning (i.e. encoding of incentive value that can then be anticipated) in these taxa (Muzio et al. 2011). From a comparative viewpoint, dogs in this study behaved less like other mammal species, but more like pigeons (Papini and Dudley 1997), toads (Papini et al. 1995), turtles (Papini and Ishida 1994) and goldfish (Lowes and Bitterman 1967) in SNC paradigms by maintaining a higher duration of eye gaze following the downshift—but this is not to say that dog does not respond emotionally.

Dogs’ behaviour in the test could also be explained by habituation: the amount of food dogs could obtain within a very short time period was very large. It is known that animals will habituate to one particular food type, which thus loses its reinforcer effectiveness with repeated presentation (McSweeney 2004). Thus, although dogs in the shifted condition may have experienced a downshift of reward, unlike the unshifted dogs they would not yet have been habituated to the dry food, which may thus have carried higher reinforcement value for them. This interpretation is supported by the fact that food rejections hardly ever occurred in the first six trials, but became more common from trial 7 onwards.

Given the suggestion that strength of SNC effect may serve as an indicator of mood, and thus animal welfare (Burman et al. 2008), it was predicted that rescue dogs would show a more pronounced SNC effect than owned dogs, due to experiencing an environment that can result in poor welfare and negative affect (Hennessy et al. 1998; Coppola et al. 2006; Taylor and Mills 2007). However, neither population showed an SNC effect, and owned and rescue dogs showed few differences in their behaviour throughout the study—although there were suggestions that rescue dogs differentiated more strongly between the high- and low-quality rewards, which is in line with the notion that individuals from poorer environmental conditions should be more sensitive to reward quality (e.g. van der Harst and Spruijt 2007). Overall, these results could indicate that there was little difference in affective state between the two populations investigated in this study, or, that any negative affective state induced by the rescue environment was ‘cancelled out’ by a rebound response to the positive aspects of the cognitive task itself, e.g. the opportunity to work for treats (Burman et al. 2011). However, it is more likely that the paradigm used here was not suitable, and some particulars of the methodology, and specifically the social aspect, may potentially account for the lack of an observed SNC effect in either population.

Following reward downshifts, animals will often show an increase in search behaviour and exploration (Flaherty 1982, 1999; Freidin et al. 2009). Dogs will naturally gaze into human faces when faced with an insoluble problem (Miklósi et al. 2003; Marshall-Pescini et al. 2013) and show gazing at the owner and gaze alternation when trying to elicit food or toys from the owner (Gaunet 2008, 2010). Accordingly, it is possible that our subjects maintained eye gaze at the experimenter in an attempt to elicit the previously obtained reward, thereby confounding any potential reduction in eye gaze as a consequence of the reward downshift. Future studies in dogs should therefore assess SNC effects on food quality/quantity using a paradigm that reduces the potential for a social confound (i.e. that does not rely upon direct interaction between the subject and the experimenter), as has been done in other animal species.

Of interest for future studies may be the use of a within-subject design, an approach that is unusual in the SNC literature (but see studies by Shettleworth and Nevin 1965; Baltzer and Weiskrantz 1970 on behavioural contrast). Using this approach has both advantages and drawbacks. We obtained quite different results in the between- and the within-subjects analysis, with treatment differences in gaze behaviour revealed for all three test phases (pre-shift, post-shift and re-shift) using a within-subjects analysis, whereas a between-subjects analysis revealed a treatment difference only during the re-shift phase. This can be explained by a higher degree of sensitivity to treatment effects by avoiding confounding effects of individual differences, leading to statistical greater power (see also Keren and Lewis 2014). However, a potential drawback of a within-subjects design is the lack of independence between treatments (Keren and Lewis 2014). We addressed this by counterbalancing the number of subjects that received the shifted and the unshifted condition first and by including the effect of test order in the models. Indeed, our analysis revealed that test order did affect performance in all three phases of the experiment: dogs that received the shifted condition first generally showed a higher level of response throughout the test. It is possible that by initially receiving a high value reward for performing the eye gaze response, those dogs that received the shifted condition first developed a more positive association with the task itself and so continued to show a high level of response—even when reward value was reduced. In other words, previous experience with the high value reward appeared to increase motivation to perform the task, divorced from the absolute value of the incentive. It is therefore suggested that future studies include additional familiarisation sessions with the food rewards, as well as with the operant task itself, prior to testing.

## Conclusions

Despite its larger sample size and the benefit of a within-subjects design that was found to give greater sensitivity and statistical power, the current study did not replicate the finding that dogs exhibited a successive negative contrast effect following a reward downshift in the eye gaze task (Bentosela et al. 2009) in either owned or rescue dogs. On the contrary, the within-subjects analysis revealed that dogs showed a reverse SNC effect by maintaining a higher level of responses following a reward downshift. Thus, SNC effects do not appear to easily replicate in a range of environmental contexts and populations outside of the laboratory, at least in domestic dogs.
